# Exercise induced anaphylaxis in kiwi allergic patient: case report

**DOI:** 10.1186/s13223-021-00595-6

**Published:** 2021-09-08

**Authors:** Natalia Ukleja-Sokołowska, Robert Zacniewski, Kinga Lis, Magdalena Żbikowska-Gotz, Andrzej Kuźmiński, Zbigniew Bartuzi

**Affiliations:** Department of Allergology, Clinical Immunology and Internal Medicine, Ludwik Rydygier Collegium Medicum in Bydgoszcz, Nicolaus Copernicus University in Toruń, ul. Ujejskiego 75, 85-168 Bydgoszcz, Poland

**Keywords:** Kiwi, Allergy, Food dependent exercise induced anaphylaxis, ImmunoCap ISAC, IgE

## Abstract

**Background:**

An allergy to kiwi is rare in Poland. Most (65–72%) of the patients who are allergic to kiwi report symptoms of an oral allergy syndrome (OAS); however, systemic manifestations (18–28%) have also been reported.

**Case report:**

A 27-year-old male patient, previously not suffering from chronic diseases, exercised in the gym. He began with isometric training and then continued with aerobic exercise on a treadmill. After exercise, he ate 2 kiwi (*Actinidia deliciosa)* fruits. He experienced a swelling of the lips after eating the fruit, followed by an itchy scalp and a swollen face. Approximately 60 min later, the symptoms worsened: the patient suffered from generalized hives, general weakness and a "rumbling" sensation in ears. The patient's condition improved upon the consumption of antihistamines. However, the swelling of the face persisted for 24 h despite previously eating a kiwi without any side effects. By means of diagnostics based on allergen components, an allergy to grass allergen components, especially timothy grass—Phl p 1, Phl p 2 and Phl p 5, was confirmed. The presence of IgE that is specific for Act d 2 kiwi was also found. The patient had an oral food challenge with kiwi fruit at rest and after exercise provocation test. The challenge was negative at rest and positive after exercise. A food-dependent exercise-induced anaphylaxis gathered with a kiwi sensitization was diagnosed.

**Conclusion:**

To our knowledge, this case is the first report of a kiwi-allergic patient in whom exercise was a necessary cofactor to induce an anaphylactic reaction.

## Introduction

In Poland, an allergy to kiwi has not been subjected to epidemiological studies. In a study carried out by Bedoll-Pulido et al. in Mexico, it was found that among 264 patients allergic to inhalant allergens, 6.6% had positive skin tests for kiwi, while only 2 (0.76%) displayed clinical symptoms [1]. To date, an exercise-related allergy to kiwi has not been reported. Food-dependent exercise-induced anaphylaxis (FDEIA) is a distinct form of allergy in which clinical symptoms occur when exposure to the allergen is accompanied with exercise. It can occur before or after an exposure to the sensitizing protein [2].

The prevalence of FDEIA is increasing in recent years. Male cases are more frequent than female. In addition, it can occur both in children and adults. Oral food challenge (OFC) followed by physical exercise (OFCPE) should be considered as the diagnostic gold standard of FDEIA [3, 4].

Kiwi was considered a non-native fruit to many developed countries during the eighties and nineties. However, it is now commonly consumed globally. In a study conducted in Finland, it was found that kiwi was in the list of top ten most allergenic foods among students [5]. An allergy to kiwi is usually associated with an allergy to grass and birch pollen. An allergy to kiwi is also common in latex-allergic patients. Most (65–72%) patients who are allergic to kiwi report symptoms in the form of oral allergy syndrome (OAS, recently addressed mostly as pollen food allergy syndrome, PFAS); however, systemic manifestations (18–28%) have also been reported [6].

Thirteen allergenic components of kiwi have been characterized. A possibility of the list of important allergens being longer in the future cannot be ruled out. The kiwi allergens that are known so far are presented in Table 1.

**Table 1:** Kiwi allergens, based on www.allergen.org

Allergen	Protein family	Molecular weight
Act d 1	Cysteine protease (actinidin)	30 kDa
Act d 2	Thaumatin-like protein	24 kDa
Act d 3		40 kDa
Act d 4	Phytocystatin	11 kDa
Act d 5	Kiwellin	28 kDa
Act d 6	Pectin methylesterase inhibitor	18 kDa
Act d 7	Pectin methylesterase	50 kDa
Act d 8	PR-10, Bet v 1 family member	17 kDa
Act d 9	Profilin	14 kDa
Act d 10	nsLTP1	10 kDa
Act d 11	Major latex protein/ripening-related protein (MLP/RRP), Bet v 1 family member	17 kDa
Act d 12	Cupin, 11S globulin	50 Da
Act d 13	2S albumin	11 Da

The main kiwi allergen recognized as a marker of allergy to this fruit is Act d 1 [7]. The contents of major allergens in an individual kiwi fruit depend on the variety of the fruit. The basic proteins of its green variety are actinidin and kiwelin. Hoffman-Sommergruber et al. showed that the Hayward variety (the so-called dark green) was the most allergenic. It was found that anaphylaxis occurred most frequently following the consumption of a fruit of this variety [8]. An interesting study published by Gavrovic-Jankulovic et al. found that the concentration of allergens also depended on the time of harvest. The highest concentration of Act c 1 and Act c 2 occurred in fruits harvested in November, and the lowest concentration was found in September [9].

Among kiwi allergens, the Bet v 1 homologues also play an important role. These may be responsible for the development of cross-allergy symptoms in patients allergic to pollen allergens mainly in the form of OAS. The Act d 11 is the first member of the MLP/RRP protein family to be described as an allergen [10].

A fruit allergy is very often associated with an allergy to latex in the form of latex-fruit syndrome. Hev b 11 and Hev b 6 latex chitinases are responsible for the occurrence of cross-reactions with kiwi [11, 12].

A cross-reactivity was detected between an isolated kiwi allergen with a molecular weight of 24 kDa and pollen of meadow fescue [13]. Cross-reactions between the Ole e 3 olive pollen allergen and kiwi have also been demonstrated [14]. Moreover, it is also assumed that other olive allergens, such as Ole e 9, Ole e 10, might play an important role in cross-reactions with the kiwi fruit [15]. Erricson et al. studied 20 patients with bronchial asthma who were allergic to wheat flour. It was found that 7 (35%) patients reacted with the OAS syndrome to consumption of kiwi fruit. This indicates a cross-reaction between the Act d 2 kiwi allergen and bromelain—one of wheat allergens. All the examined patients were positive for specific IgE for the standard kiwi fruit extract and for specific IgE for Act d 1 and Act d 2 allergen proteins, which were detected in more than half (57 and 43%) of the tested sera. A cross-reactivity was confirmed using an ELISA-inhibition test. The inhibition of IgE binding was over 50% in cases of wheat and kiwi extracts [16].

This paper presents the use of food and exercise provocation tests in an FDEIA patient that was developed following the consumption of kiwi.

## Case report

A 27-year-old male patient was admitted to the Department of Allergology, Clinical Immunology and Internal Medicine for the diagnosis of food allergy in April 2018. The patient was not suffering from chronic diseases and was not taking any regular medication. For a duration of 3 years prior to admission, he had reported a runny nose as well as itching of the nose and eyes occurring in spring.

The symptoms related to food allergy appeared in January 2018. The patient exercised in the gym, initially undergoing isometric training and then aerobic exercise on a treadmill. After exercise, he ate 2 kiwi (*Actinidia deliciosa)* fruits. There was lip swelling within 5 min after eating the fruit, followed by an itchy scalp and a swollen face. Approximately 60 min later, the symptoms worsened: the patient suffered from generalized hives, general weakness and a "rumbling" sensation in ears. The patient's condition improved within 25 min of consuming antihistamines, but the swelling of the face persisted for 24 h. The patient had previously eaten kiwi without any issues. The patient denied any other contributing factors which potentially could have enhanced an allergic reaction, such as excessive stress, alcohol or NSAID consumption. On the day of allergic reaction he ate a regular breakfast, but cannot remember the specific foods he consumed.

The patient was not a smoker. He occasionally consumed alcohol. He lived alone and worked as a welding foreman. The patient's grandfather was receiving treatment for bronchial asthma.

During hospitalization, a detailed history of the patient's symptoms was taken. The patient's physical examination displayed no abnormalities. Several diagnostic tests were performed, including skin prick tests that were negative for rye flour, wheat flour, barley flour, orange, clementine, banana, potato, pepper, cod, carp, chicken egg, cow's milk, pork, beef and meat II. However, the skin prick tests were positive with grass, rye, weed and *Dermatophagoides pteronyssinus* house dust mite allergens (Allergopharma). The skin prick by a prick test with fresh kiwi fruit was positive. The patient underwent a bronchial challenge with histamine, and the result was within the range of norm.

Blood was collected from the patient for assessing the levels of specific IgE antibodies against the selected inhalant and food allergens using the highly sensitive ImmunoCap method. The results are summarized in Table 2.

**Table 2:** Results of immunological tests

Total IgE	92.6 kU/l
Levels of specific IgE in ImmunoCap (kU/L)	
* Dermatophagoides pteronyssinus*	< 0.35
* Dermatophagoides farinae*	< 0.35
Dog	< 0.35
Early grass	43.45
Late grass	17.68
Mugwort	< 035
Kiwi	0.94
Shrimp	1.71

Based on the medical history, symptoms reported by the patient and test results, the patient was diagnosed with allergic rhinitis associated with an allergy to timothy grass. At the same time, the test results showed a presence of IgE that is specific for kiwi and shrimp allergens. Although the patient had an elevated concentration of shrimp sIgE, he had no allergy symptoms after consuming shrimp. The consumption of kiwi was associated with an anaphylactic reaction that occurred after exercise. The patient repeatedly consumed kiwi and presented no symptoms. Considering the inconsistency between the medical history and results of the laboratory tests, a decision was made to perform a provocation test.

On the first day, the patient attempted to perform an exercise provocation on an empty stomach by warming up on a bicycle ergometer for 10 min at a speed leading to heart rate of 120 bpm after 4 min of exercise. The actual exercise challenge was performed on a treadmill with continuous monitoring of the heart rate at 80%–90% of the maximum heart rate (HR max) for 10 min. The patient completed the trial without displaying any side effects during and after exercise. On the second day, the patient consumed 2 ripe, peeled kiwi fruits in incremental doses in the monitored room. The patient did not present any adverse effects during the 24-h observation period.

On the next day, the patient ate a kiwi fruit. Following 30 min of observation, an exercise challenge was performed according to the protocol described above. Five minutes after the exercise, the patient developed abdominal pain, skin lesions in the form of urticarial on the skin of the face and dry cough. A physical examination revealed single wheezing over the lung fields, heart rate of 80 bpm and blood pressure of 117/82 mmHg. A 0.5 mg dose of adrenaline was administered intramuscularly as well as intravenous steroids and antihistamines were given. All ailments resolved quickly.

The investigation was extended by determining the levels of allergenic components using the ImmunoCap ISAC method. The results are shown in Table 3.

**Table 3:** Results of the ISAC ImmunoCap test

Allergen group	Allergen source	Allergen component	ISAC standardized units (ISU-E)
Grass pollen	Bermuda grass	cCyn d 1	3.3
	Timothy grass	rPhl p 1	20
		rPhl p 2	8.1
		rPhl p 5	13
		rPhl p 6	0.5
Thaumatin-like protein	Kiwi	nAct d 2	1.8

The table includes all allergen components with an elevated level of specific IgE

Range

< 0,3 Undeterminable

0,3–0,9 Low

1–14,9 Medium/High

>  = 15 Very high

By means of the diagnostics based on allergen components, an allergy to grass allergen components, especially timothy grass—Phl p 1, Phl p 2 and Phl p 5, was confirmed. The presence of IgE specific for Act d 2 present in kiwi was also found.

The patient was discharged with the following diagnosis: Exercise-induced anaphylaxis, food-dependent and allergic rhinitis.

The patient was directed to start allergen-specific immunotherapy with grass allergens. In addition, he was also recommended to remove kiwis from his diet. Owing to the fact that many fruits and vegetables can potentially be a cause of cross reaction due to Act d 2 homologues, caution was recommended when ingesting fruits and vegetables for 4 h prior to and 2 h following exercise. Due to a possible, unexpected anaphylaxis, especially in relation to exercise, alcohol or NSAIDS. An epinephrine auto-injector was recommended to be carried on an everyday bases. The patient was educated in the management of anaphylaxis.

For six months after hospitalization, the patient did not experience anaphylaxis and upon a follow up visit, was advised to continue to follow previous recommendations.

## Discussion

An allergy to kiwi may have a variable clinical course, ranging from benign skin lesions, through local oral symptoms to life-threatening anaphylaxis. The course of the reaction depends on various factors. This includes general health, an allergen's sensitizing potential, the amount of the allergen to which the patient is exposed and additional factors that may enhance the course of the reaction. The described patient tolerated significantly large amounts of the allergen at rest and ate 2 kiwi fruits without complications in the provocation test. However, at times, even a small amount of kiwi can cause reactions. An interesting case of a patient developing life-threatening anaphylaxis upon the consumption of a minimal amount of kiwi allergens was presented in 2013. The patient suffered an anaphylactic shock after consuming vanilla ice cream, which was served with a spoon previously used for kiwi ice cream. Anaphylaxis also appeared in this patient during the allergology diagnostics triggered by skin prick test with a fresh kiwi fruit [17].

The present case is considered to be interesting mainly because the anaphylactic reaction occurred typically as a post-exercise reaction. FDEIA, which was diagnosed in a patient, is best described for lipid transfer protein (LTP) allergens and for wheat flour omega-5-gliadin allergy. In cases of described allergies, the diagnosis is based on the analysis of the history and a precise determination of the sensitizing protein. Exercise and food provocation are the final indications used to confirm the diagnosis. However, it should be used with caution, as it may lead to an anaphylactic reaction [18, 19].

To our present knowledge, this case is the first report of a kiwi-allergic patient in whom exercise was a necessary cofactor to induce an anaphylactic reaction. The patient did not experience any allergic symptoms to kiwi at rest. Other cofactors that may worsen the course of allergic reactions are alcohol and aspirin. Christensen et al. demonstrated that alcohol and aspirin were sufficient to induce a response in patients who were diagnosed with wheat dependent exercise induced anaphylaxis (WDEIA) [20].

Exercise, alcohol, NSAIDs, etc. are cofactors of an allergic reaction. The European Academy of Allergy and Clinical Immunology (EAACI) Taskforce on Anaphylaxis defines cofactors as patient-related or external circumstances that are associated with increased severe allergic reactions. These are also known as augmentation factors [21].

There are several theories on the manner in which cofactors enhance allergic reaction. Evidence suggests that an increased permeability of gastrointestinal track after exercise or alcohol ingestion is associated with increased exposition to food allergens. Other evidence suggests an independent mechanism promoting mast cell degranulation or the influence of increased osmolality of blood serum. The exact mechanism is still under investigation [22].

The ImmunoCap ISAC demonstrated that among all kiwi allergens, the patient was only allergic to Act d 2. It is a thaumatin-like protein (TLPs) found in various edible plants. They belong to the PR family (pathogenesis related) and have a molecular weight of 20–30 kD and a compact 3-dimensional structure held together by 8 disulfide bridges. TLPs are characterized by a resistance against digestion in the digestive tract and are insensitive to acids and high temperature. They are also present in food after cooking or industrial processing [23, 24].

In 2014, Azofra García et al. described the first case of recurrent anaphylaxis in a 31-year-old female patient, after eating various taxonomically unrelated foods, including banana, grapes, red wine, tomatoes, apples, garlic, carrots, lettuce and onions. The patient tolerated kiwi. The immunological tests confirmed the presence of TLP-specific IgE from various sources, including Act d 2, and skin tests showed positive results for purified TLP and Act d 2 [25]. In case of the patient presented in this study, anaphylaxis appeared only after kiwi combined with exercise, but in the pathogenesis of anaphylaxis, an allergy due to Act d 2 seems to be a probable causal factor. On the other hand, participation of other kiwi allergens in the development of anaphylaxis in the patient cannot be ruled out. The ImmunoCap ISAC test contains only four kiwi allergen components—Act d 1, Act d 2, Act d 5 and Act d 8. It is possible that the patient is also allergic to an allergen component not included in this test, such as Act d 10 that belongs to the LTP family.

The nsLTP (non-specific lipid transfer proteins) are plant panallergenes that are clinically relevant mainly as food allergens but also described in plant pollen. An LTP allergy, initially thought to mainly exist in southern Europe, is becoming accepted as a cause of allergic reactions to plant foods across many countries around the world. The peach nsLTP allergen Pru p 3 is a dominant sensitizing allergen. Peaches are a common food trigger, although multiple foods can be involved. In particular, those belonging to the *Rosacea* family—peach (Pru p 3), apple (Mal d 3) can be involved, but also the *Corylaceae* family (Latin Corylus) e.g. in hazelnuts (Cor a 8); the *Juglandaceae* family e.g. in walnuts (Jug r 3); the *Fabaceae* family, e.g. peanuts (Ara h 9) and many others [26–28]. Some patients tolerate peeled fruits and vegetables due to the fact that LTP is mainly found in the peel [29]. A clinical manifestation of allergy may significantly differ even in the same patient, and a significant intensification of symptom severity occurs especially in the presence of co-factors [30, 31]. Kiwi LTP is Act d 10, a minor allergen, with the weight of 10 kDa; it has homologous sequences to the allergens present in apricot (Pru ar 3), mulberry (Mor n 3), peach (Pru p 3), orange (Cit s 3), peanut (Ara h 9) and hemp pollen (Can s 3) [32]. In the described patient, it is unlikely that LTP was the source of symptoms. Although there is no Act d 10 allergen component available in ImmunoCap ISAC, there is a number of other cross-reacting nsLTP, and all of them are negative.

Another interesting aspect in this case is that although the patient had positive shrimp sIgE, his history did not align with the shrimp allergy. Shrimp allergy is also associated with FDEIA. Owing to lack of any allergy-related symptoms, the patient does not require any form of shrimp avoidance solely based on elevated level of shrimp sIgE [33].

During the diagnosis, the allergy to wheat flour, including omega-5-gliadin, was excluded based on the interview, skin prick tests and ImmunoCap ISAC result. Nevertheless, omega-5-gliadin sensitization is worth mentioning during the differential diagnosis due to a common manifestation of FDEIA—wheat dependent exercise induced anaphylaxis (WDEIA). Sensitization to house dust mite is also a possible factor of exercise induced anaphylaxis, especially after ingesting food containing dust mite-contaminated flour. Dust mite ingestion-associated, exercise-induced anaphylaxis may be misdiagnosed as WDEIA and should be suspected in patients with an anaphylaxis linked to food intake and exercise but also in those who have no apparent evidence to the index food ingredients [34]. The described patient had positive SPT with Dermatophagoides pteronyssinus, but negative sIgE with house dust mite. No sIgE specific to Dermatophagoides pteronyssinus allergen components were found in ImmunoCap ISAC and the symptoms reported by the patient do not align with HDM sensitization. Therefore, this diagnosis seems unlikely.

Diagnosis in the patient was largely based on provocation tests. A double-blind, placebo-controlled food challenge remains the gold standard of diagnosis in food allergy. However, owing to the tests being time-consuming, expensive and carrying the risk of an adverse reaction in a patient, this type of diagnostics is relatively rare. However, there are cases when they are necessary. Despite the increased availability of many diagnostic methods, including those based on allergen components, a non-invasive and generally available method that would allow determination whether symptoms will develop following an exposure to a given allergen. If such is the case the knowledge of its extent remains unavailable [35]. Oral provocation tests are especially indicated in patients for the following reasons: for whom it is unclear what product caused the allergic reaction; in assessing whether the tolerance of a given product has been achieved; following the use of allergen-specific immunotherapy, when effectiveness of the treatment needs to be established; when extension of the diet in a patient with polyvalent allergy who uses an elimination diet is planned and to exclude or confirm allergy in patients whose allergy test results are inconsistent with the history of reactions [36–39]. A consideration of co-factors of an allergic reaction in order to strengthen the course of the reaction or induce it in a patient who did not have symptoms at rest is yet a separate problem. This type of diagnostics is solely used in highly specialized centers by experienced research teams. There are no specific guidelines on the methodology of such provocation tests, and the available knowledge is based on case reports and single studies on larger populations, mainly conducted in patients allergic to wheat omega-5-gliadin [20, 40].

In the reported case, the provocation test was of open character, which was sufficient for the diagnosis. The open provocation test is the easiest to perform. A negative result of an open provocation test indicates no hypersensitivity to the tested food, while a positive result is diagnostic, provided that objective symptoms occur. The development of subjective unclear symptoms indicates the need for a blinded test [41]. The preparation of a blinded sample requires substantive preparation on the part of the examination team, as well as devoting time and resources to the correct preparation of the test. Obtaining a sample containing a placebo and an active sample of the same consistency, appearance and taste is difficult [41]. Following the open test, the patient had objective symptoms in the form of urticarial on the skin of the face, as well as dyspnea and wheezing over the lung fields. The test result was clear and the symptoms met the criteria for anaphylaxis.

This demonstrates that patients need to be under continuous supervision by trained professionals during the challenge, which is usually performed in a hospital setting [36].

It is also extremely important to carefully select patients for testing in a proper manner, to approach each case individually and to refrain from provocation test when the risk of anaphylaxis is greater than the potential benefit of the test.

It is difficult to estimate the prognosis for a patient with an FDEIA diagnosis. In theory, life-threatening allergic reactions are not expected if the combination of ingesting the allergenic food and exercise is avoided. On the other hand, co-factors of allergic reactions may unexpectedly appear in the life of patient. The threshold of allergen, which could provoke anaphylaxis, may also decrease in the future. Patients' safety is a priority and epinephrine auto-injector is a mandatory safety measure in all cases that are at risk of anaphylaxis.

## Conclusion

This is the first reported patient to have an allergy to kiwi associated with food-induced exercise-induced anaphylaxis (FDEIA). In establishing the diagnosis, it was necessary to perform an oral food challenge with a cofactor in the form of physical exercise. Immunological diagnostics allowed the demonstration of sensitization to the allergenic component of kiwi—Act d 2. However, the possibility that sensitization to other allergen components of kiwi could be related to these symptoms cannot be ruled out.

## Data Availability

The dataset supporting the conclusions of this article are included within the article.

## Abbreviations

IgE: Immunoglobulin E
sIgE: Specific Immunoglobulin E
